# Hinweise und Empfehlungen für die Konzeption von Lehrkräftefortbildungen zu digitalen Medien

**DOI:** 10.1007/s11618-021-01046-z

**Published:** 2021-09-27

**Authors:** Lukas Schulze-Vorberg, Claudia Krille, Sabine Fabriz, Holger Horz

**Affiliations:** Arbeitsbereich Pädagogische Psychologie, Goethe-Universität Frankfurt am Main, Theodor-W.-Adorno-Platz 6, 60629 Frankfurt am Main, Deutschland

**Keywords:** Digitalisierung, Latente Profilanalyse, Lehrkräftefortbildung, Fortbildungsgestaltung, TPACK, ICT, Latent profile analysis, Teacher professional development, TPACK

## Abstract

Um Unterricht durch digitale Medien lernwirksam gestalten zu können, sollten Lehrkräfte über die notwendigen Kompetenzen für einen didaktisch angemessenen und reflektieren Einsatz von Technologien verfügen. Neben der Verbesserung der technischen Infrastruktur an Schulen ist es daher notwendig, Lehrkräfte bei ihrer Professionalisierung zu unterstützen. Lehrkräfte an deutschen Schulen zeigen allerdings eine eher zurückhaltende Teilnahme an Fortbildungsangeboten zu digitalen Themen, was auf eine fehlende Passung zu den realen Bedarfen hinweisen kann. Der vorliegende Beitrag widmet sich daher Präferenzen von Lehrkräften zu Inhalten und Gestaltungsmerkmalen von Fortbildungen zu digitalen Medien und berichtet dazu Ergebnisse einer Befragung von Gymnasiallehrkräften (*N* = 238). Um möglichst zielgruppenspezifische Ergebnisse zu erhalten, wurden über eine latente Profilanalyse mit Personenmerkmalen (technologisches und technologisch-pädagogisches Wissen, Selbstwirksamkeit, Mediennutzung) drei Profile identifiziert und im Hinblick auf ihre Präferenzen verglichen. Die Ergebnisse weisen auf eine Vielfalt an thematischen Wünschen sowie auf die Notwendigkeit einer bedarfsgerechten Gestaltung von Fortbildungen hin. Abschließend werden zusammenfassende Empfehlungen zur Gestaltung von Lehrkräftefortbildungen zu digitalen Medien formuliert.

## Abstract (en)

For a reflective and purposeful use of information and communication technology (ICT) in and out of the classroom, school teachers need to be equipped with the necessary knowledge and skills. In addition to improving the technical infrastructure in schools, opportunities for support need to be provided. However, teachers at German schools have shown low participation rates in professional development activities concerning ICT related teaching and learning, which may indicate a lack of alignment with real needs. This article addresses teachers’ preferences for topics and organizational design criteria of teachers’ professional development regarding ICT use and reports results of a survey with secondary school teachers (*N* = 238). To obtain an information base for tailored results for the target group, a latent profile analysis utilizing technological and technological pedagogical knowledge, self-efficacy, and media use, yielded three profiles. The profiles were compared in their preferences for professional development. Results revealed a wide range of proposed topics by the teachers as well as the need for differential design of teacher professional development regarding ICT. Recommendations for the conceptual design of professional development programmes for teachers on the use of digital media are presented.

## Einleitung

Durch die zunehmenden Digitalisierungsprozesse werden Kompetenzen im Umgang mit digitalen Medien für die gesellschaftliche Teilhabe relevanter. Folgerichtig werden diese in den Empfehlungen und Strategien zur Integration digitaler Medien in Bildungsprozesse von der Kultusministerkonferenz (KMK 2016) als vierte Kulturtechnik genannt. Der kompetente Umgang mit digitalen Medien wird dabei in Anlehnung an bestehende digitalisierungsbezogene Kompetenzrahmen (z. B. DigComp, Ferrari 2013) durch nicht getrennt voneinander zu betrachtende Kompetenzbereiche, wie beispielsweise Kommunizieren und Kooperieren, Problemlösen und Handeln oder Analysieren und Reflektieren, verstanden (KMK 2016).

Im Kontext des schulischen Lernens mit digitalen Medien sind Lehrkräfte daher zum einen gefordert, Schülerinnen und Schülern Fertigkeiten zum Einsatz und die kompetente Nutzung von digitalen Medien zu vermitteln. Zum anderen werden sie auch vor die Herausforderung gestellt, digitale Medien lernförderlich in der Entwicklung und Umsetzung von neuen Formen des Unterrichtens (z. B. Hillmayr et al. 2017; Klieme 2020) und der Verbesserung des fachlichen Lernens zur berücksichtigen (Eickelmann und Gerick 2018).

Die bereits vor der COVID-19-Pandemie bekannten flächendeckenden Nachholbedarfe in den Bereichen der schulischen Digitalisierung und des technologisch-pädagogischen Lehrkräftewissens (z. B. Drossel et al. 2019) zeigten sich während der pandemiebedingten Schulschließungen besonders deutlich: Fehlende digitale Schulkonzepte und die unzureichende Vorbereitung von Lehrkräften auf digitale Unterrichtsszenarien wurden sichtbar (z. B. Eickelmann und Gerick 2020). Gleichzeitig stellte sich das technologisch-pädagogische Wissen der Lehrkräfte als ein wichtiger Faktor für eine erfolgreiche Adaptation an die Herausforderungen des virtuellen Unterrichtens heraus (König et al. 2020). Eine aktuelle Ländervereinbarung der KMK (2020) adressiert diese Bedarfe, indem beispielsweise die Verankerung fachdidaktischer Kompetenzen zur Nutzung digitaler Medien in Curricula der Lehramtsausbildung, aber auch die Bereitstellung fächer- und stufenübergreifender digitaler Lehr- und Lernmittel bis 2025 besonders betont werden. Als weiterer zentraler Aspekt wird dort der Aufbau und die stetige Aktualisierung der Qualifizierung von Lehrkräften in digitalisierungsbezogenen Kompetenzen über alle Phasen der Lehrkräftebildung hinweg genannt.

Im Hinblick auf die geplanten, fortschreitenden schulischen Digitalisierungsprozesse (z. B. DigitalPakt Schule, Bundesministerium für Bildung und Forschung, BMBF 2019) birgt gerade die dritte Phase der Lehrkräftebildung im Sinne eines lebenslangen Lernens ein großes Potenzial für eine kontinuierliche berufliche Begleitung von Lehrkräften im Umgang mit den sich ständig ändernden Herausforderungen und Potenzialen digitaler Medien in Schule und Unterricht (z. B. Eickelmann und Drossel 2020a; Herzig und Grafe 2007; Lorenz und Endberg 2019; Walitzek 2021). In dieser Phase können zum einen Lehrkräfte erreicht werden, die bislang kaum systematisch mit der Thematik in Berührung kamen, zum anderen auch Personen angesprochen werden, die bestehende Kenntnisse vertiefen und erweitern möchten (Eickelmann und Drossel 2020a).

Dass es möglich ist, die (selbsteingeschätzte) Kompetenz zum Einsatz digitaler Medien im Schulkontext über Lehrkräftefortbildungen auf- und auszubauen, bestätigt die einschlägige Forschung vielfältig (z. B. Alt 2018; Chai et al. 2019; Koh et al. 2017). Auch zeigen Befunde zu Lehrkräftefortbildungen, dass insbesondere die Teilnahme an sorgfältig gestalteten Fortbildungsveranstaltungen günstige Effekte auf die Leistung von Schülerinnen und Schülern haben kann (z. B. Lipowsky und Rzejak 2019; Timperley et al. 2007). Allerdings liegen Studienergebnisse vor, denen zufolge bundesweit bisher nur ein geringer Anteil von Lehrkräften an digitalisierungsbezogenen Fortbildungen teilnahm (Gerick et al. 2019). Es ist allerdings zu diesen Befunden kritisch anzumerken, dass – wie in den meisten Studien zur Fortbildungsteilnahme (Richter 2016) – das verfügbare Fortbildungsangebot nicht in den Blick genommen wurde (Gerick et al. 2019). Ein immer wieder von Lehrkräften angeführter Grund für das Fernbleiben von Fortbildungen ist die fehlende Passung von Fortbildungsangeboten und den von ihnen geäußerten Bedarfen (z. B. Büsching und Breiter 2011; Gagarina und Saldern 2010) oder die Sicht der Lehrkräfte, dass andere Fortbildungsthemen wichtiger sind (BITKOM 2015; Büsching und Breiter 2011; Gallasch et al. 2000).

Da intrinsische Faktoren wie das Interesse von Lehrkräften im Zusammenhang mit deren Fortbildungsteilnahme und -auswahl steht (z. B. Krille 2020), kann eine hohe Passung zwischen dem Fortbildungsangebot und ihren Bedarfen oder Wünschen die Bereitschaft zu einer Teilnahme erhöhen. Zwar liegen Studien zu Wünschen hinsichtlich Fortbildungsthemen vor (z. B. Kammerl et al. 2016), jedoch sollten solche Ergebnisse aufgrund des raschen digitalen Wandels regelmäßig erneuert werden. Neuere Ergebnisse liegen nur aggregiert auf Schulebene vor (z. B. in der International Computer and Information Literacy Study 2018; Gerick et al. 2019) und werden eher breit zusammengefasst berichtet. Hierdurch werden zwar ein Überblick sowie das Aufzeigen von Trends ermöglicht, es bleibt jedoch offen, welche konkreten Themen in Fortbildungsprogrammen aufgegriffen werden sollten. Den Studien ist gemein, dass Themen unabhängig von der Fortbildungsgestaltung erfragt wurden, wobei letztere auch mit der wahrgenommenen Attraktivität (z. B. Krille 2020) sowie der Wirksamkeit (z. B. Lipowsky und Rzejak 2019) zusammenhängt.

Daher sollte die Perspektive von Lehrkräften berücksichtigt werden, um durch sorgfältig und zielgruppenspezifisch konzipierte Fortbildungen die Teilnahmewahrscheinlichkeit in der anvisierten Zielgruppe zu erhöhen. Der Beitrag widmet sich daher der Frage, welche Präferenzen Lehrkräfte zu Fortbildungen zum Einsatz digitaler Medien äußern und wie sich diese Wünsche vor dem Hintergrund bestehender Befunde zu Fortbildungen einordnen lassen. Über den Einbezug von individuellen Variablen wie dem technologisch-pädagogischen Wissen und dem Medieneinsatz im Unterricht sollen weiterhin Profile identifiziert werden, die einen Einblick in systematische Zusammenhänge mit Fortbildungswünschen erlauben.

## Lehrkräftefortbildungen zu digitalen Medien

### Von Lehrkräften gewünschte Fortbildungsinhalte

Hinweise zu möglichen Schwerpunktthemen für Fortbildungen zum Einsatz digitaler Medien und Präferenzen von Lehrkräften lassen sich aus Lehrkräftebefragungen zu bereits besuchten Fortbildungen ableiten. In ICILS 2018 gab jeweils rund ein Drittel der befragten deutschen Lehrkräfte an, in den letzten zwei Jahren Fortbildungen zur Integration digitaler Medien in Lehr- und Lernprozesse oder zu fächerspezifischen Inhalten besucht zu haben (Gerick et al. 2019). Etwa ein Viertel nahm an Kursen zu Anwendungsprogrammen teil. Nur ein Bruchteil (rund 5 %) gab an, Kurse zur Nutzung digitaler Medien durch Schülerinnen und Schüler mit sonderpädagogischem Förderbedarf besucht zu haben. Diese Angaben sind nicht unabhängig vom verfügbaren Angebot zu sehen, doch liefern sie bereits Hinweise darauf, welche inhaltliche Fortbildungsgestaltung in diesem Bereich als wichtig von der Zielgruppe empfunden wird.

Ebenso können Befragungsergebnisse zu Fortbildungswünschen herangezogen werden: Im *Länderindikator 2016* wurden unter anderem offene Antworten von 453 Lehrkräften zu Fortbildungswünschen hinsichtlich des Einsatzes digitaler Medien ausgewertet (Kammerl et al. 2016). Es wurden sechs Bereiche identifiziert, wobei sich gut ein Viertel (25,7 %) der Wünsche auf Fortbildungen zum Einsatz digitaler Medien im Unterricht oder für Hausaufgaben bezogen. Weitere 21,6 % nannten den Umgang mit digitalen Medien, dem Internet, sozialen Netzwerken oder persönlichen Daten, 17,9 % den Umgang mit Software und 14,0 % der Lehrkräfte führten die computergestützte Förderung von Schülerinnen und Schülern an. Jeweils rund 10 % nannten den Umgang mit Hardware bzw. den Einsatz digitaler Medien für Schulverwaltung und -organisation. Hervorzuheben ist hier der deutliche Bedarf an didaktischen Einsatzszenarien, die in der Vergangenheit einem Überangebot an technischen und anwendungsbezogenen Fortbildungen für Lehrkräfte gegenüberstanden (vgl. Gerick und Eickelmann 2017). Da bisher nur wenige Studien den Fokus auf Fortbildungswünsche auf Veranstaltungen zu digitalen Medien fokussieren, erscheint es sinnvoll, auch Erkenntnisse aus breiter ausgerichteten Studien zu berücksichtigen: Allgemein betrachtet wünschen sich Lehrkräfte vor allem solche Veranstaltungsinhalte, die einen klaren Bezug zum Unterrichtsalltag sowie zu ihren unterrichteten Fächern haben, und die darüber hinaus leicht umsetzbaren Konzepte vermitteln und den Austausch mit anderen Lehrkräften ermöglichen (Krille 2020).

### Präferierte Gestaltungsmerkmale von Fortbildungen aus Lehrkräftesicht

Neben den Inhalten einer Fortbildung spielen auch deren Gestaltungsmerkmale eine wichtige Rolle für ihre Wirksamkeit, zum Beispiel in Form von veränderten Lehrkräftekognitionen, Handeln im Unterricht oder Lernerfolg von Schülerinnen und Schülern (z. B. Lipowsky 2010; Lipowsky und Rzejak 2019). Diese Gestaltungsmerkmale umfassen strukturelle Merkmale, wie zeitliche Bedingungen und die Organisation, sowie Lernaktivitäten der Teilnehmenden und die Expertise der Referentinnen und Referenten (Lipowsky 2010). Dem erweiterten Angebots- und Nutzungsmodell für Fortbildungsveranstaltungen von Lipowsky (2010) zufolge beeinflussen diese Merkmale unter anderem die Wahrnehmung und Nutzung des Fortbildungsangebots durch die Lehrkräfte. Für Empfehlungen zur Gestaltung von Fortbildungen sollten also auch Befunde aus Sicht von Lehrkräften berücksichtigt werden, die jedoch bisher noch wenig systematisch beforscht sind (Richter 2016). Meist werden diese nur indirekt – beispielsweise über die Angabe von Hinderungsgründen für Fortbildungsteilnahmen – abgefragt. Betrachtet man diese Ergebnisse, so zeigt sich, dass die von Lipowsky (2010) zusammengetragenen Gestaltungsmerkmale auch aus Lehrkräftesicht für eine Fortbildungsteilnahme relevant sind: Neben der Länge und dem Zeitpunkt einer Fortbildungsveranstaltung sind dies auch Aspekte wie der Veranstaltungsort (und die Anreise zu diesem), der Teilnehmendenkreis, die Expertise der Dozierenden sowie die erwarteten Aktivitäten während der Veranstaltung (Krille 2020).

Eine umfangreiche Studie, die neben den Wünschen zu Fortbildungsthemen auch deren Ausgestaltung in den Blick nahm, wurde 1989 durchgeführt und liegt damit mehr als 30 Jahre zurück (Reckmann 1992). Allerdings wurden die Gestaltungsmerkmale nicht im Zusammenhang mit den Inhalten betrachtet, da die Untersuchung keinen inhaltlichen Themenschwerpunkt hatte. Es gibt allerdings Hinweise darauf, dass die Präferenzen von Gestaltungsmerkmalen von verschiedenen Aspekten abhängen (z. B. dem Inhalt einer Fortbildung oder dem Interesse der Lehrkraft an dieser Thematik; Krille 2019; Riedel et al. 1994).

Um Anhaltspunkte für Gestaltungsmerkmale von Fortbildungen zu erhalten, können auch Befunde zu den Wirkfaktoren von Lehrkräftefortbildungen berücksichtigt werden (Desimone 2011; Lipowsky und Rzejak 2019). Allerdings stehen die aus den empirischen Studien abgeleiteten Empfehlungen zum Teil den Bedürfnissen der Lehrkräfte entgegen: Fortbildungen konkurrieren potenziell mit Unterrichtsaufgaben oder auch anderen pragmatischen Gründen, wie beispielsweise der Kinderbetreuung am Nachmittag (Krille 2020). Passend dazu zeigen die Ergebnisse von Richter et al. (2020), dass mehrheitlich halbtägige (Einzel‑)Veranstaltungen die Fortbildungslandschaft dominieren. Doch solche zeitlichen Voraussetzungen stellen dahingehend eine besondere Herausforderung dar, da zu kurze Fortbildungen nur wenig Gelegenheit für aktive Partizipation oder den Transfer auf den eigenen Unterricht bieten (z. B. Desimone 2011; Lipowsky und Rzejak 2019). Es muss berücksichtigt werden, dass die hier angesprochenen Gestaltungsmerkmale für Fortbildungen eher im Sinne von unterrichtlichen „Sichtstrukturen“ zu verstehen sind (vgl. z. B. Kunter und Trautwein 2013). Für den Erfolg von Lerngelegenheiten – zum Beispiel Unterricht oder Fortbildungen – sind allerdings insbesondere die sogenannten „Tiefenstrukturen“ relevant, also unter anderem die aktive Auseinandersetzung mit dem Gelernten durch Anwendungsmöglichkeiten und Reflexionsphasen (z. B. Lipowsky und Rzejak 2019). Die Gestaltungsmerkmale bilden daher eine wichtige Rahmenbedingung dafür, dass eine solche Auseinandersetzung mit den Fortbildungsinhalten überhaupt stattfinden kann.

### Personenmerkmale im Kontext von digitalisierungsbezogenen Fortbildungen

Es ist davon auszugehen, dass in der Präferenz für bestimmte Inhaltsbereiche auch Variablen der Lehrpersonen eine Rolle spielen. Es zeigte sich zum Beispiel, dass männliche Lehrkräfte bei Fortbildungsveranstaltungen mit eher technischen Themen überrepräsentiert waren (Krille 2020) beziehungsweise das weibliche wie auch ältere Lehrkräfte eher ablehnende Haltungen zur Medienkompetenzförderung vertreten (Kammerl 2018). Sieve (2015) zeigte darüber hinaus, dass Lehrkräfte mit einer höheren Selbsteinschätzung der Fähigkeiten im Umgang mit digitalen Tafeln eher an Fortbildungen zu dieser Thematik teilnahmen. Auch themenunspezifisch gibt es Hinweise darauf, dass die Selbstwirksamkeitserwartung der Lehrkräfte mit der Teilnahme an Fortbildungen prinzipiell aber auch mit der Auswahl von einem breiteren Themenspektrum zusammenhängt (Richter et al. 2013). Darüber hinaus zeigte sich, dass Lehrkräfte, die ein größeres Vorwissen zu digitalen Medien haben – und sich entsprechend bereits mit der Thematik auseinandergesetzt haben – eher dazu bereit sind, sich neues Wissen in diesem Bereich anzueignen (z. B. Herzig und Grafe 2007; Kammerl 2018). Dieses Ergebnis deckt sich mit den Überlegungen der sogenannten Neigungshypothese (z. B. Richter 2011), nach der Lehrkräfte vor allem Fortbildungsthemen in Bereichen auswählen, die sie bereits im Studium vertieft haben. Auch zeigte eine Analyse von Drossel et al. (2019) zu Professionalisierungstypen, dass die identifizierte (größte) Gruppe der zurückhaltenden Professionalisierer, die eher selten pädagogische und technologische Professionalisierungsmaßnahmen in Anspruch nehmen, besonders selten angaben, die digitalen Kompetenzen ihrer Schülerinnen und Schüler zu fördern. Entsprechend sollten auch bei der Untersuchung von Präferenzen der Lehrkräfte hinsichtlich Fortbildungsthemen im Bereich digitaler Medien individuelle Merkmale wie (technologisches) Wissen oder die Medienintegration in den Unterricht berücksichtigt werden.

Ein häufig in Forschung und Praxis verwendetes Modell zur Beschreibung und als Grundlage zur Erfassung (z. B. Baier und Kunter 2020; Schmidt et al. 2009; Schulze-Vorberg 2021) von technologiebezogenen Wissensbereichen bei Lehrkräften ist das *Technological Pedagogical Content Knowledge*-Modell (TPACK) von Mishra und Koehler (2006). Das an Shulmans (1986) Modell des Professionswissens angelehnte TPACK-Modell erweitert die beiden Kern-Wissensbereiche des inhaltlichen Wissens (CK: *content knowledge*) und pädagogischen Wissens (PK: *pedagogical knowledge*) sowie deren Schnittmengen um die Facette des technologischen Wissens (TK: *technological knowledge*). Dieses umfasst sowohl das Wissen über bereits etablierte Medien wie Tafel und Whiteboard, als auch vor allem das Wissen über neue technologiebasierte Medien sowie das in diesem Kontext relevante Wissen zur Informationsverarbeitung, Kommunikation und Problemlösung. Insbesondere die Schnittmenge von TK und PK (technologisch-pädagogisches Wissen, TPK) soll hervorgehoben werden, da dieses die Kenntnis über die Möglichkeiten verschiedener Technologien und deren sinnvoller Einsatz in Lehr-Lernszenarien beschreibt (Koehler und Mishra 2009). TPK sollte dabei auch Wissen zu einem pädagogisch sinnvollen Umgang mit technologiebasierten Problemsituationen mit einbeziehen, um eine möglichst hohe aktive Lernzeit bei Schülerinnen und Schülern sicherstellen zu können.

Studien zu TPACK und digitalisierungsbezogenen Variablen konnten zeigen, dass hohe Ausprägungen auf den Wissensfacetten des TPACK-Modells einen positiven Zusammenhang mit weiteren digitalisierungsbezogenen Kompetenzaspekten wie der digitalen Selbstwirksamkeit oder der empfundenen Nützlichkeit digitaler Medien, aufweisen (z. B. Joo et al. 2018). Der Nachweis eines direkten Zusammenhangs mit dem beabsichtigten Einsatz von digitalen Technologien in Unterrichtskonzepten konnte in jüngsten Studien allerdings nicht erbracht werden (z. B. Backfisch et al. 2020; Schmid et al. 2021).

### Fragestellungen

Ausgehend von dem hohen Potenzial von Lehrkräftefortbildungen für den Bereich digitaler Medien im Schul- und Unterrichtskontext sollten Angebote entwickelt werden, die sowohl eine hohe Qualität durch Evidenzbasierung gewährleisten als auch in Inhalten und Rahmenbedingungen mit den Anforderungen der Zielgruppe korrespondieren. Hierfür sollen in der vorliegenden Studie bereits existierende Befunde zu möglichen Themenwünschen erneuert werden. Darüber hinaus soll die bisher bestehende Forschungslücke von Präferenzen von Lehrkräften hinsichtlich verschiedener Gestaltungsmerkmale spezifisch für den Kontext von Fortbildungen zu digitalen Medien durch die Untersuchung adressiert werden.

Wie dargestellt, ist davon auszugehen, dass insbesondere Vorwissen und Vorerfahrungen im Einsatz digitaler Medien in Schule und Unterricht sowie motivationale Variablen die Auswahl von Fortbildungsveranstaltungen mitbestimmen. Daraus resultierende Profile sollten für die Entwicklung von Fortbildungen wie auch für die Ansprache der Zielgruppe von Relevanz sein. Hieraus ergeben sich für die vorliegende Studie zwei leitende Forschungsfragen:

1. Welche (a) thematischen Wünsche und (b) Präferenzen zur organisatorischen Gestaltung von Fortbildungen zu digitalen Medien werden von Lehrkräften genannt?
2. Unterscheiden sich Lehrkräfte mit unterschiedlichen Ausprägungen in technologisch-pädagogischem Wissen, digitalem Medieneinsatz und Selbstwirksamkeit im Hinblick auf Wünsche zu Inhalten und Präferenzen zur Gestaltung von Fortbildungen zu digitalen Medien?
  - Ergeben sich aus den digitalen Wissensbereichen (TK, TPK), dem digitalen Medieneinsatz und der Selbstwirksamkeit zum Unterrichten mit digitalen Medien von Lehrkräften Profile, die sich sinnvoll interpretieren lassen?
  - Lassen sich zur Validierung der Profile unter Berücksichtigung der Neigungshypothese folgende Hypothesen bestätigen: Profile mit hohen Ausprägungen auf den in Forschungsfrage 2a genannten Variablen weisen eine signifikant (1) höhere Anzahl an besuchten Fortbildungen zu digitalen Medien auf und (2) geben seltener als Hinderungsgrund für den Besuch einer solchen Veranstaltung mangelndes Interesse an.
  - Inwieweit unterscheiden sich die etwaigen Profile bezüglich den Hinderungsgründen sowie Fortbildungswünschen und Präferenzen zur organisatorischen Gestaltung von Lehrkräftefortbildungen zu digitalen Medien?

## Methode

### Stichprobe

Über elektronische und postalische Anschreiben wurden 34 mit der Goethe-Universität kooperierende Schulen im Raum Frankfurt über die Ansprache der Schulleitungen eingeladen, an einer Erhebung als Grundlage zur Erstellung eines Medienbildungskonzeptes und weiteren Forschungszwecken teilzunehmen. Die Teilnahme erfolgte computergestützt über die Software Unipark, teilweise synchron im Rahmen von pädagogischen Tagen aber auch asynchron über einen personalisierten Einladungslink via E‑Mail. Von Februar bis April 2020 nahmen insgesamt 238 Gymnasiallehrkräfte (133 weiblich; Berufserfahrung *M* = 13,58 Jahre, *SD* = 8,84) von sieben Schulen teil. Insgesamt 217 der Lehrkräfte gaben an, Fächer aus dem sprachlich-literarisch-künstlerischen zu unterrichten, 112 nannten Fächer aus dem gesellschaftswissenschaftlichen und 139 Fächer aus dem mathematisch-naturwissenschaftlich-technischen Aufgabenfeld, 31 Lehrkräfte gaben an, Sport zu unterrichten und 15 Nennungen konnten weiteren Fächern zugeordnet werden. Die befragten Lehrkräfte gaben an, in den letzten zwei Jahren im Mittel rund sechs (*M* = 5,8, *SD* = 6,29) Fortbildungen besucht zu haben, wovon sich im Durchschnitt ein bis zwei (*M* = 1,6, *SD* = 2,65) Fortbildungen auf den Umgang mit oder den Einsatz von digitalen Medien bezogen.

### Instrumente

#### Themenwünsche und präferierte Gestaltungsmerkmale

In Anlehnung an die Studie von Reckmann (1992) wurden die Lehrkräfte gebeten, zwei Fortbildungsveranstaltungen hinsichtlich digitaler Medien sowie deren Merkmale zu nennen, an denen sie gern teilnehmen würden. Für jede gewünschte Veranstaltung sollten sie zunächst in einem offenen Antwortformat den Fortbildungsinhalt und anschließend im geschlossenem Antwortformat die präferierte Gestaltung anhand verschiedener Merkmale (siehe Tab. 4) auswählen. Die Zusammenstellung dieser Merkmale geschah dabei auf Basis von Reckmann (1992) sowie dem Angebots-Nutzungs-Modell von Lipowsky (2010).

#### Hinderungsgründe

Zudem konnten die Lehrkräfte in einem 5‑stufigen Rating (1 = *trifft nicht zu *bis 5 = *trifft zu*) angeben, wie stark potenzielle Hinderungsgründe (21 Items, angelehnt an Lenski et al. 2016) für die Teilnahme an Schulungen zu digitalen Medien in den letzten 12 Monaten für sie zutrafen (Tab. 3).

#### Technologisches (pädagogisches) Wissen

Das technologische (sieben Items, α = 0,91, *M* = 3,46, *SD* = 0,84) und das technologisch-pädagogische Wissen (neun Items, α = 0,86, *M* = 3,48, *SD* = 0,67) der Lehrkräfte wurde über eine adaptierte Selbsteinschätzungsskala auf einer fünfstufigen Likertskala (1 = *stimme überhaupt nicht zu* bis 5 = *stimme voll und ganz zu*) erhoben (Schmidt et al. 2009).

#### Der kompetente Umgang mit digitalen Problemsituationen im Unterricht

Zur objektiven Erfassung (eines Teilbereichs) des TPK, des Umgangs von Lehrkräften mit digitalen Problemsituationen im Unterricht, wurde ein *Situational Judgment Test* (Schulze-Vorberg 2021) genutzt. In diesem eindimensionalen Test werden Lehrkräften in insgesamt 13 kurzen Vignetten digitale Problemsituationen im Unterricht dargeboten (z. B. benötigte Software lässt sich im Unterrichtsverlauf nicht nutzen) und die Beurteilung der Passung von je fünf bis sieben Handlungsalternativen auf einer sechsstufigen Skala (1 = *sehr kompetent* bis 6 = *überhaupt nicht kompetent*) erfragt. Bei Übereinstimmung der Bewertung der Handlungsalternative mit einem durch Expertinnen und Experten ermittelten Modalwert wurde je ein Punkt vergeben (maximal 73 Punkte, α = 0,81, *M* = 20,71, *SD* = 7,72).

#### Digitale Selbstwirksamkeitserwartung

Zur Erfassung wurde eine adaptierte Fassung der Selbstwirksamkeitserwartungsskala (SWK) von Schwarzer und Jerusalem (1999) eingesetzt. Die adaptierte Skala umfasst zehn Items auf einer vierstufigen Likertskala (1 = *stimmt nicht* bis 4 = *stimmt genau*) und bezieht sich auf die Selbstwirksamkeit bei der digitalen Mediennutzung im Unterricht (α = 0,93, *M* = 2,67, *SD* = 0,84).

#### Tägliche Nutzung digitaler Medien

Diese wurde über die Angabe der Mediennutzung (Computer, Notebook, Tablet, Digitalkamera, interaktives Whiteboard, Beamer, Visualizer, Smartphone) auf einer 5‑stufigen Skala erhoben (1 = *jeden Tag *bis 5 = *nie*). Die Angaben wurden zur vereinfachten Analyse analog zum Vorgehen bei ICILS dichotomisiert (Drossel et al. 2019). Wenn mindestens ein digitales Medium täglich im Unterricht genutzt wurde, wurde das Item mit eins kodiert, bei allen anderen Angaben wurde eine Null vergeben.

### Analysen

#### Inhaltsanalyse zur Entwicklung des Kategoriensystems für die inhaltlichen Themenwünsche

Zu den gewünschten Fortbildungsthemen konnten insgesamt 437 Antworten (*n* = 39 fehlend oder nicht auswertbar) berücksichtigt werden. Anhand von rund 10 % der Angaben wurde zunächst induktiv ein Kodierschema mit 22 Kategorien entwickelt, dabei wurden Beispiele generiert und Kodierregeln erstellt. Die Antworten wurden im nächsten Schritt deduktiv in die Kategorien von Kammerl et al. (2016) eingeordnet und für eine detaillierte Betrachtung in weitere Teilaspekte differenziert (Tab. 1). Alle Antworten der Lehrkräfte wurden von zwei unabhängigen geschulten Beurteilenden mit Unterstützung des entwickelten Kodiermanuals kodiert. Die Beurteilungsübereinstimmung lässt sich als sehr gut einschätzen (κ = 0,83; Döring und Bortz 2016). Im Falle einer Nichtübereinstimmung fand im Diskurs eine Entscheidung für eine Zuordnung der Antwort statt.

**Table 1:** 

					**%**		
	Kategorie	Ankerbeispiele	*N*	Ges	DA	DM	DV
***Computergestützte Förderung der SuS***							
1	Differenzierung mithilfe digitaler Medien	Individuelle Förderung	7	1,6	0,0	1,5	3,8
***Einsatz digitaler Medien im Bereich Schulverwaltung und -organisation***							
2	Unterrichtsplanung & -monitoring mit digitaler Software	Digitale Unterrichtsplanung	16	3,7	2,0	4,3	3,8
***Einsatz digitaler Medien (Computer, Tablet, Smartphone etc.) im Unterricht oder für Hausaufgaben***							
3	Grundlagen digitaler Mediennutzung	Vermittlung von Grundkenntnissen basierend auf der Schulausstattung	95	21,7	31,4	18,3	19,7
4	Einsatz digitaler Unterrichtsmaterialien	Arbeiten mit digitalen Schulbüchern	17	3,9	2,0	5,1	2,6
5	Erstellung digitaler Produkte	Erklärvideos erstellen	30	6,9	4,0	7,4	8,8
6	Einsatz von Lernsoftware/Apps/Digitalen Tools	Apps für den Fremdsprachenunterricht	47	10,8	9,0	10,6	13,8
7	Arbeiten mit Lernplattformen	Aufbau einer Lernplattform für SuS	32	7,3	9,9	6,6	6,2
8	Kooperation mithilfe digitaler Medien	Digitale Zusammenarbeit	10	2,3	4,0	2,3	0,0
9	Digitales Prüfen	Mediengestützte Lernkontrollen, wie z. B. *Bettermarks*	3	0,7	1,0	0,8	0,0
10	Einsatz v. Smartphones	Einsatz von Smartphones der SuS	5	1,1	0,0	1,2	2,4
11	Einsatz v. Tablets	Tablets im Fremdsprachenunterricht	38	8,7	8,9	9,3	6,1
12	Einsatz v. Laptop/Computer	Einsatz von SuS-Laptops	3	0,7	2,0	0,4	0,0
13	Umgang mit Problemsituationen beim Einsatz digitaler Medien	Verhalten bei technischen Problemen	8	1,8	1,0	1,9	2,5
***Umgang mit digitalen Medien, Internet, sozialen Netzwerken oder persönlichen Daten***							
14	Vermittlung von Medienkompetenz	Medienerziehung: Umgang mit Internetquellen	18	4,1	2,0	5,9	1,2
15	Herausforderungen für Lehrkräfte im Umgang mit digitalen Medien	Umgang mit Ablenkung der SuS durch digitale Medien	9	2,1	2,0	2,0	2,5
***Umgang mit Hardware (Beamer, Smart‑/Whiteboard etc.)***							
16	Einsatz v. Smart‑/White‑/Activeboards	Professionelles Arbeiten mit den Aktivboards	48	11,0	12,8	12,1	5,3
***Umgang mit Software (Bildbearbeitung, Grafikprogramme, Office etc.)***							
17	Umgang mit Grundlagen-software außerhalb des Unterrichts	Bildbearbeitung	19	4,3	3,1	4,3	6,0
***Sonstiges***							
18	Fachwissen (Informatik)	Programmieren mit Scratch	4	0,9	0,0	0,0	5,0
19	Erfahrungsaustausch	Austausch mit Fachkollegen an Pilotschulen	3	0,7	1,0	0,0	2,5
20	Medienausstattung	Einrichtung iPad-Klasse	8	1,8	0,0	1,9	3,9
21	Einsatz programmierbarer Taschenrechner	Programmierbarer Taschenrechner im Unterricht	2	0,5	1,0	0,8	0,0
***Nicht kategorisierbar***							
22	Nicht kategorisierbar	Ökonomie	15	3,4	3,1	3,5	3,8

*Anmerkungen. *Kursive, nicht nummerierte Zwischenüberschriften entsprechen den Kategorien von Kammerl et al. (2016)

*N* = Häufigkeit der Nennung über zwei Fortbildungswünsche hinweg, *GES* = Anteil an den Gesamtnennungen, *DA* = digital Abseitsstehende, *DM* = digital Mithaltende, *DV* = digital Vorreitende, *SuS* = Schülerinnen und Schüler

#### Latente Profilanalyse

Zur differenzierten Betrachtung der zweiten Forschungsfrage wurde eine Latent-Profile-Analyse (LPA, Muthén 2004) unter Einbezug der erhobenen Kennwerte zu technologiebezogenem Wissen (TK, TPK), digitaler Selbstwirksamkeitserwartung (SWK) und digitaler Mediennutzung durchgeführt. Da für die Bearbeitung der einbezogenen Skalen Pflichtfelder verwendet wurden, liegen keine fehlenden Werte vor. Das Ziel der LPA lag darin, Profile oder Gruppen von Lehrkräften zu identifizieren, deren Mitglieder sich in den einbezogenen Merkmalen ähneln und sich in diesen von anderen Profilen beziehungsweise Gruppen unterscheiden (Ferguson et al. 2020). In die Analysen wurden die latent modellierten Factorscores aus konfirmatorischen Faktorenanalysen (z. B. Brown 2006) der zur LPA herangezogenen Skalen genutzt. Factorscores wurden anstelle manifester Mittelwerte gewählt, da die beobachteten Antworten so gewichtet in die Berechnung eingehen und die Residuen der manifesten Items nicht inkludiert werden (Höft 2020). Die konfirmatorischen Faktorenanalysen sowie die LPA wurden mit dem Programm Mplus 6.1 durchgeführt (Muthén und Muthén 2010). Zur Evaluation der ermittelten Profile wurde eine Diskriminanzanalyse mit der Software SPSS (Version 26.0) durchgeführt. Hierfür wurde das Kreuzvalidierungsverfahren *Leave-one-out* genutzt, bei dem je ein Fall separiert wird und durch die ermittelte Funktion aus den restlichen Fällen bestimmt wird (*N*–1), bis alle Fälle einmal ausgeschlossen wurden. Dadurch kann eine große Stichprobenreduktion, wie beispielsweise bei der Aufteilung in zwei Teilstichproben, vermieden werden (Browne 2000).

#### Analyse von Gruppenunterschieden

Zur Bestätigung der aufgestellten Validierungshypothesen (Forschungsfrage 2b) zu den latenten Profilen (Geiser 2011; Spurk et al. 2020) sowie zur Prüfung, inwiefern sich diese Zielgruppen in ihren Präferenzen der Gestaltungsmerkmale unterscheiden, wurden je nach Datenniveau der abhängigen Variable multivariate Varianzanalysen (metrische Variablen, z. B. Berufserfahrung, besuchte Fortbildungen) oder Chi-Quadrat-Tests (kategoriale Variable, z. B. Präferenzen der Fortbildungsgestaltungsmerkmale) genutzt. Im Nachgang der Varianzanalysen wurden Posthoc-Tests angewendet. Aufgrund der unterschiedlichen Gruppengrößen zwischen den Profilen wurde der GT2 von Hochberg ausgewählt (Field 2018). Die Analysen wurden mithilfe der Software SPSS (Version 26.0) durchgeführt.

## Ergebnisse

(1a) Zur Beantwortung der Teilforschungsfrage zu den *thematischen Fortbildungswünschen* wurden zunächst mithilfe des oben dargestellten Kategoriensystems die beiden genannten Themenwünsche jeder Lehrkraft kategorisiert. Die deskriptiven Ergebnisse sind in Tab. 1 dargestellt. Es zeigte sich, dass insbesondere einführende Kurse zu Grundlagen der digitalen Mediennutzung als Fortbildungsthema gewünscht wurden. 10,8 % der Angaben ließen sich jeweils den Kategorien *Einsatz von Smart‑/White‑/Activeboards* und *Einsatz von Lernsoftware/Apps/Digitalen Tools im Unterricht* zuordnen. Die Erstellung von digitalen Produkten sowie das Arbeiten mit Lernplattformen wurden von 6,9 % Lehrkräften gewünscht. Zum Einsatz von Hardware durch Schülerinnen und Schüler wurden Tablets am häufigsten erwähnt, während Smartphones oder Laptops und Computer kaum eine Rolle spielten. Die Mehrheit aller Wünsche waren als fachunspezifischer Wunsch formuliert (FW1: 74,8 % FW2: 76,5 %), wobei der Fachbezug nicht explizit abgefragt, sondern lediglich aus dem Themenwunsch der Lehrkräfte abgeleitet wurde.

(1b) Zur Bearbeitung der zweiten Teilforschungsfrage wurden die Präferenzen bei der organisatorischen Gestaltung von Fortbildungen zu digitalen Medien deskriptiv ausgewertet und tabellarisch aufbereitet (Tab. 4). Hinsichtlich des *Niveaus* der Fortbildungen wünschten sich die Lehrkräfte vor allem Grundlagenkurse und weiterführende Kurse. Bezogen auf die *Expertise der Fortbildungsleitenden* wurden deutlich Lehrkräfte präferiert, die modellhaft die Praxis präsentieren können. Allerdings gaben vergleichbar viele an, dass der Hintergrund der Referentinnen oder Referenten egal wäre. Andere Personen mit thematischer Expertise wurden eher selten gewählt.

Beim geeigneten *zeitlichen Rahmen *waren ganztägige und am Vormittag stattfindende Veranstaltungen am beliebtesten. Mehrtägige Veranstaltungen und Fortbildungsreihen wurden kaum gewählt. Die Frage nach dem präferierten *Zeitpunkt* ergab für knapp zwei Drittel der Lehrkräfte eine Präferenz für *während der Unterrichtszeit*. An Kommentaren war allerdings erkennbar, dass *während des Unterrichts* sowohl in dem Sinne verstanden wurde, dass Unterricht ausfallen würde als auch im Sinne von *außerhalb der Ferien*. Im Hinblick auf den *Ort* der gewünschten Fortbildungen wurde vor allem die eigene Schule ausgewählt oder angegeben, dass es egal sei, wo die Veranstaltung stattfinde. Auch bei der Zusammensetzung des *Teilnehmendenkreis* gaben sehr viele Befragte an, dass dieser Aspekt irrelevant für sie sei.

Als geeignete *Arbeitsform* sahen die befragten Lehrkräfte vor allem die Informationsvermittlung mittels Medien und solche, in denen Unterrichtsmaterialien erarbeitet oder gesichtet und bewertet werden. Auch die Erprobung von Unterrichtskonzepten und Kleingruppenarbeit wurden als gewünschte Arbeitsformen ausgewählt. Auffällig war, dass ausschließlich online stattfindende Veranstaltungen für die Befragten am wenigsten attraktiv waren, gefolgt von Blended-Learning-Formaten.

In einer explorativen Betrachtung wurde zusätzlich ermittelt, inwiefern die Befragten die verschiedenen Veranstaltungsmerkmale an die jeweiligen Fortbildungswünsche anpassten. Hierfür wurde für jede Person bestimmt, ob es einen Unterschied zwischen dem Merkmal bei Fortbildungswunsch 1 im Vergleich zu Wunsch 2 gibt. Dabei zeigte sich, dass 20 Personen (8,4 %) keinerlei Änderungen in ihren präferierten Veranstaltungsmerkmalen vornahmen. Knapp über die Hälfte der Befragten (54,2 %) passten lediglich ein oder zwei Merkmale an. Die wenigsten änderten fünf (4,2 %) oder gar alle (1,7 %) der Merkmale.

**Figure 1:**
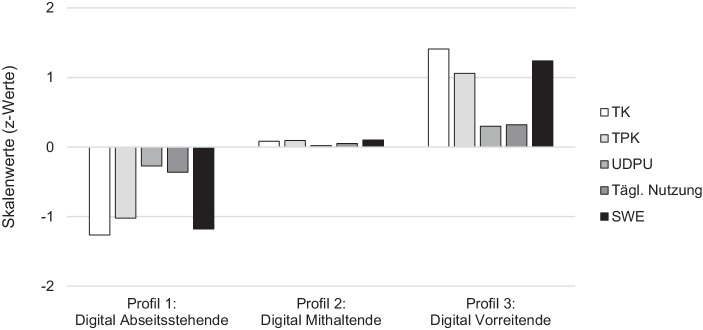

(2) Um die zweite Forschungsfrage nach Unterschieden in den Wünschen der Fortbildungsgestaltung von Lehrkräften mit verschiedenen Personenmerkmalen zu beantworten, wurden die drei Teilforschungsfragen wie folgt bearbeitet:

(2a) Die erste Teilforschungsfrage nach sinnvoll interpretierbaren Profilen von potenziellen Teilnehmenden an Fortbildungen wurde mithilfe einer LPA adressiert. Alle Teilnehmenden wurden in dieser Analyse gemäß ihren Ausprägungen der technologiebezogenen Wissensskalen (TK, TPK) und Selbstwirksamkeit (SWK) sowie der täglichen Nutzung digitaler Medien betrachtet (Tab. 2). In der Analyse wurden fünf Modelle geschätzt und bezüglich der in der Literatur häufig empfohlenen FIT-Indizes (AIC, BIC, SABIC, Entropy LMR, BLRT; Beschreibungen in Tab. 2) verglichen (Geiser 2011; Marsh et al. 2009). Wie aus Tab. 2 ersichtlich wird, nehmen die Kennwerte AIC und SABIC mit zunehmender Anzahl von Profillösungen ab. Der BIC hingegen deutet auf eine 3‑ oder 4‑Profillösung hin. Der BLRT liefert kein eindeutiges Ergebnis und weist für alle Modelle die Lösung mit einem Profil weniger als die bessere aus. Der LMRT hingegen zeigt, dass sich die 3‑ und 5‑Profillösungen gegenüber der Profillösung mit einem Profil weniger signifikant verbessert. Hinsichtlich sinnvoller Profilgrößen zeigen 5 Profile eine sehr kleine (< 1 %) und damit schwer interpretierbare Profillösung. Der Entropy-Wert jedoch stützt die 3‑Profillösung. Aufgrund der ermittelten Kennwerte, des Parsimonitätsprinzips sowie die auch bei der Auswahl zur berücksichtigende Interpretierbarkeit der Profile wurde die 3‑Profillösung gewählt (vgl. Geiser 2011). Die drei ermittelten Profile, die sich terminologisch an den Nutzertypen des D21 Digital-Index (Initiative D21 e. V. 2020) orientieren (digital Vorreitende, digital Mithaltende, digital Abseitsstehende), unterscheiden sich dabei substanziell in den betrachteten Merkmalen (Abb. 1).

**Table 2:** 

Profile	Anzahl der Parameter	AIC	BIC	SABIC	Entropy	*p* LMR	BLRT	< 1 %
2	16	2075,909	2131,465	2080,750	0,717	0,099	0,000	0
3	22	1945,220	2021,610	1951,877	0,860	0,000	0,000	0
4	28	1924,229	2021,452	1932,701	0,793	0,127	0,000	0
5	34	1911,302	2029,360	1921,590	0,831	0,001	0,004	1

*Anmerkungen*. *N*= 238; *AIC* = Akaike Information Criterion, *BIC* = Bayesian Information Criterion, *SABIC* = Sample size adjusted BIC, *p LMR* = Lo-Mendell-Rubin likelihood ratio test und *BLRT* = Bootstrap-Likelihood-Ratio-Differenzentest für *n* (H0) vs. *n*-1 Profile; < 1 % zeigt die Anzahl der ermittelten Profile, die weniger als 1 % der Stichprobe umfassen

Im Hinblick auf die Geschlechterverteilung zeigen Chi-Quadrat-Tests, dass ein Zusammenhang der Profile und dem Geschlecht besteht (χ^2^(4) = 17,28, *p* = 0,002): Weibliche Lehrkräfte waren bei den digital Abseitsstehenden (70,4 %) und den digital Mithaltenden (60,9 %) deutlich häufiger vertreten, als bei den digital Vorreitenden (34,1 %). Die drei Profile unterscheiden sich zudem signifikant (*F*(2, 235) = 7,71, *p* = 0,001) in der Berufserfahrung. Die Posthoc-Analyse zeigt, dass die digital Abseitsstehenden die höchste Berufserfahrung aufweisen (*M* = 17,43, *SD* = 8,56) und sich damit signifikant von den anderen beiden Gruppen unterscheiden (Mithaltende: *M* = 12,37, *SD* = 8,33, *p* = 0,001; Vorreitende: *M* = 12,23, *SD* = 9,44, *p* = 0,008).

**Figure 2:**
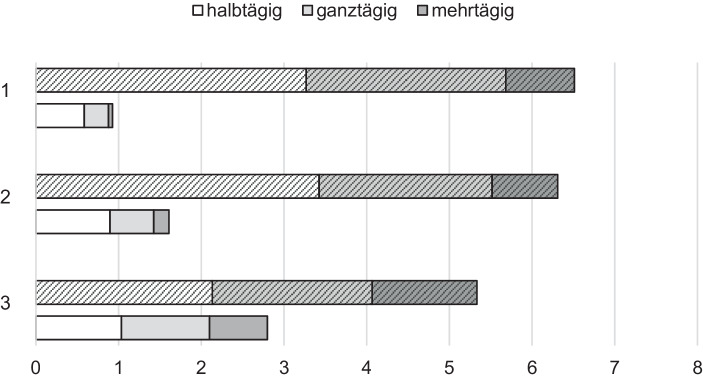

In der Evaluation der 3‑Profillösung mit einer* Leave-one-out*-Kreuzvalidierung konnten zwei Funktionen ermittelt werden (*p* < 0,001, Wilks *λ* = 0,199; *p* = 0,89, Wilks *λ* = 0,885), durch die 95 % der ursprünglich kodierten Fälle und 93,7 % der kreuzvalidierten Fälle korrekt klassifiziert werden konnten.

(2b) Zur Überprüfung der Validität der ermittelten Profile wurden Varianzanalysen mit den Profilen als unabhängige und der Anzahl besuchter Fortbildungen im Bereich digitaler Medien sowie dem Hinderungsgrund zur Fortbildungsteilnahme aufgrund mangelnden Interesses an der Thematik als abhängige Variablen herangezogen. Es konnte ein signifikanter Unterschied zwischen den Profilen bei den besuchten Fortbildungsveranstaltungen in den letzten zwei Jahren gezeigt werden (Abseitsstehende: *M* = 0,93, *SD* = 1,79; Mithaltende: *M* = 1,61, *SD* = 3,05; Vorreitende: *M* = 2,80, *SD* = 3,66; *F*(2, 155) = 3,60, *p* = 0,030, η = 0,044). Dabei unterscheidet sich die Anzahl der besuchten Fortbildungen signifikant zwischen den Abseitsstehenden und den Vorreitenden (*p* = 0,025, Abb. 2). Hervorzuheben ist, dass sich dies nur bei medienbezogenen Fortbildungen, nicht aber bei insgesamt besuchten Fortbildungen zeigte (*F*(2, 155) = 0,25, *p* = 0,776). Beim fehlenden Interesse an der Arbeit mit digitalen Medien (Abseitsstehende: *M* = 2,86, *SD* = 1,31; Mithaltende: *M* = 1,89, *SD* = 1,00; Vorreitende: *M* = 1,26, *SD* = 0,64; *F*(2, 201) = 28,33, *p* < 0,001, η = 0,22) unterscheiden sich alle drei Gruppen signifikant voneinander (*p* < 0,005).

(2c) Um zu untersuchen, ob sich die gefundenen Profile hinsichtlich angegebener Hinderungsgründe für Fortbildungsteilnahmen sowie ihrer Präferenzen bei Gestaltungsmerkmalen von Fortbildungsveranstaltungen zu digitalen Medien unterscheiden, wurden für alle erhobenen Merkmale jeweils Gruppenunterschiede mithilfe von Chi-Quadrat-Tests geprüft.

### Hinderungsgründe für Fortbildungsteilnahme

Bei den Gründen für eine Nicht-Teilnahme an medienbezogenen Fortbildungen (Tab. 3) zeigten sich keine signifikanten Unterschiede in den eher allgemeinen Hindernissen (z. B. fehlende Angebote, ausgebuchte Kurse, unpassende Zeiten, Sorge wegen Unterrichtsausfall, negative Erfahrungen in der Vergangenheit, zu hohe Kosten oder persönliche Gründe). Hinsichtlich themenspezifischer Gründe zeigte sich zunächst, dass insbesondere den Abseitsstehenden Angebote für Anfängerinnen und Anfänger im Umgang mit digitalen Medien fehlen. Umgekehrt stimmten Vorreitende und Mithaltende stärker zu, dass das verfügbare Angebot zu stark Grundlagen vermittelte. Auch bei weiteren Hinderungsgründen stimmten die Abseitsstehenden stärker zu als ihre Kolleginnen und Kollegen aus den anderen beiden Gruppen: Hierzu gehörten, dass andere Themen für Fortbildungen wichtiger waren und ein Einsatz von digitalen Medien im Unterricht nicht für die Zukunft vorgesehen war. Die Mithaltenden und Vorreitenden stimmten im Vergleich zu den Abseitsstehenden signifikant häufiger zu, dass sie nicht an Fortbildungen teilnahmen, weil sie sich auch ohne Fortbildungen auf dem Laufenden halten könnten. Sowohl die Abseitsstehenden als auch die Mithaltenden standen dem verfügbaren Angebot kritischer gegenüber und stimmten zu, dass dieses keinen Bezug zur Schulrealität hätte.

**Table 3:** 

Wenn ich mich in den letzten 12 Monaten dagegen entschieden habe, an Fortbildungen zum Einsatz digitaler Medien in der Schule teilzunehmen, dann lag das daran, dass …	Gesamt	DA	DM	DV	MANOVA ^a^			
	*M* (*SD*)	*M* (*SD*)	*M* (*SD*)	*M* (*SD*)	*F*	*p*	η	Post-hoc ^b^
… ich die Fortbildungsinhalte aufgrund der schlechten technischen Ausstattung meiner Schule nicht anwenden konnte	2,61 (1,55)	2,90 (1,50)	2,62 (1,51)	2,18 (1,49)	2,49	0,085	0,024	–
… ich keine für mich geeigneten Fortbildungsangebote fand oder kannte	3,13 (1,36)	3,25 (1,32)	3,07 (1,35)	2,95 (1,47)	0,59	0,555	0,006	–
… die interessanten Angebote zu schnell ausgebucht waren	2,00 (1,18)	1,92 (1,20)	2,05 (1,11)	1,92 (1,30)	0,32	0,730	0,003	–
… die verfügbaren Angebote nicht für AnfängerInnen geeignet waren	1,91 (1,08)	2,41 (1,22)	1,89 (1,00)	1,32 (0,77)	**12,59**	**0,000**	**0,111**	1 > 2 > 3
… die verfügbaren Angebote nur Grundlagen vermittelten, aber nicht für fortgeschrittene AnwenderInnen geeignet waren	2,07 (1,15)	1,61 (0,83)	2,12 (1,06)	2,58 (1,52)	**8,53**	**0,000**	**0,078**	1 > 2 = 3
… die angebotenen Fortbildungen keinen ausreichenden fachdidaktischen Bezug hatten	2,61 (1,36)	2,63 (1,31)	2,57 (1,32)	2,63 (1,55)	0,06	0,946	0,001	–
… mir andere Themen für Fortbildungen wichtiger waren	3,51 (1,42)	4,04 (1,20)	3,44 (1,35)	2,97 (1,64)	**6,85**	**0,001**	**0,064**	1 > 2 = 3
… ich kein Interesse an der Arbeit mit digitalen Medien hatte	2,00 (1,16)	2,86 (1,31)	1,89 (0,99)	1,26 (0,64)	**28,33**	**0,000**	**0,220**	1 > 2 > 3
… die verfügbaren Angebote meist keinen Bezug zur Schulrealität hatten	2,65 (1,26)	3,10 (1,19)	2,65 (1,23)	2,08 (1,24)	**7,59**	**0,001**	**0,070**	1 = 2 > 3
… ich zukünftig ohnehin keine digitalen Medien im Unterricht einsetzen will	1,50 (0,90)	2,04 (1,13)	1,38 (0,76)	1,21 (0,74)	**13,15**	**0,000**	**0,116**	1 > 2 = 3
… die Angebote zu unpassenden Zeiten stattfanden	2,90 (1,35)	2,82 (1,32)	2,94 (1,33)	2,89 (1,47)	0,13	0,879	0,001	–
… es schwierig war, ausfallenden Unterricht durch eine andere Lehrkraft abzudecken	2,54 (1,40)	2,33 (1,41)	2,55 (1,35)	2,74 (1,55)	0,92	0,400	0,009	–
… ich aus den Fortbildungen in der Regel wenig praktischen Nutzen zog	2,62 (1,24)	2,76 (1,18)	2,57 (1,21)	2,47 (1,37)	0,68	0,509	0,007	–
… ich in bisherigen Fortbildungen wenig Neues gelernt habe	2,38 (1,13)	2,22 (0,88)	2,40 (1,17)	2,42 (1,24)	0,55	0,575	0,005	–
… ich mich ohne Fortbildungen auf dem Laufenden halten konnte	2,62 (1,26)	1,94 (1,05)	2,75 (1,17)	2,95 (1,49)	**10,03**	**0,000**	**0,091**	1 > 2 = 3
… meine Erfahrungen mit Fortbildungen bislang eher enttäuschend waren	2,47 (1,24)	2,43 (1,08)	2,39 (1,23)	2,50 (1,41)	0,11	0,893	0,001	–
… ich manchmal einfach nicht die Energie zur Teilnahme hatte	2,76 (1,30)	2,90 (1,32)	2,75 (1,31)	2,50 (1,35)	1,02	0,364	0,010	–
… die indirekten Kosten (Reisekosten, Verpflegung etc.) zu hoch waren	2,16 (1,31)	2,04 (1,26)	2,19 (1,26)	2,32 (1,51)	0,50	0,607	0,005	–
… ich generell selten an (zusätzlichen) Fortbildungen teilnahm	2,35 (1,25)	2,25 (1,13)	2,43 (1,26)	2,16 (1,37)	0,85	0,429	0,008	–
… sich meine Schule nicht an den Kosten für Fortbildungen beteiligte	2,00 (1,17)	2,25 (1,29)	1,97 (1,10)	1,84 (1,24)	1,54	0,216	0,015	–
… es oft schwierig war, eine Kinderbetreuung zu organisieren	2,17 (1,51)	1,98 (1,39)	2,39 (1,58)	1,87 (1,40)	2,39	0,094	0,023	–

*Anmerkungen. *Signifikante Ergebnisse sind fettgedruckt.

*DA* = digital Abseitsstehende (1), *DM* = digital Mithaltende (2), *DV* = digital Vorreitende (3)

^a^ df = 2, 201

^b^ Basierend auf GT2 von Hochberg

### Fortbildungsgestaltung

Die prozentualen Verteilungen über die drei Profile können den Tab. 1 und 4 entnommen werden. Da die Ergebnisse im Zusammenhang mit den Wünschen zur Fortbildungsgestaltung sehr umfangreich sind, wird im Folgenden vor allem auf auffällige Befunde eingegangen. Im Hinblick auf die gewünschten *Fortbildungsthemen* (Tab. 1) ist auffällig, dass sich die Gruppe der Abseitsstehenden besonders häufig Inhalte wünschte, die als *Grundlagen digitaler Mediennutzung* kategorisiert werden können, im Vergleich zu den anderen beiden Gruppen. Sie wünschten sich außerdem häufiger Fortbildungen zum Arbeiten mit Lernplattformen. Sowohl Abseitsstehenden als auch die Mithaltenden nannten häufiger Fortbildungswünsche im Zusammenhang mit dem Einsatz von Tablets sowie von Smart‑/White‑/Activeboards im Vergleich zu den Vorreitenden. Wünsche hinsichtlich der Erstellung digitaler Produkte sowie dem Einsatz von Lernsoftware/Apps im Unterricht wurden von den Abseitsstehenden seltener genannt als in den anderen beiden Gruppen. Statistisch signifikante Unterschiede ergaben sich allerdings nur für die Themen des ersten Fortbildungswunsches (χ^2^(42) = 61,72, *p* = 0,03), während sich diese nicht beim zweitgenannten Wunsch zeigten (χ^2^(40) = 48,49, *p* = 0,17).

**Table 4:** 

	Gesamt		DA		DM		DV	
	FW 1	FW 2	FW 1	FW 2	FW 1	FW 2	FW 1	FW 2
*Niveau (****χ***^***2***^***(4)*** ***=*** ***45,71*, *p*** ***<*** ***0,001*** *| ****χ***^***2***^***(4)*** ***=*** ***33,15*,* p*** ***<*** ***0,001****)*								
Einstiegs- oder Grundlagenkurs	53,6	55,7	81,0	81,0	50,0	55,2	27,3	26,2
Weiterführender oder vertiefender Kurs	39,2	36,7	17,2	17,2	45,6	39,6	47,7	57,1
Expertenkurs	7,2	5,9	1,7	1,7	4,4	5,2	25,0	16,7
*Expertise der Referenten (χ*^*2*^*(8)* *=* *9,92, p* *=* *0,271 | χ*^*2*^*(8)* *=* *9,72, p* *=* *0,285)*								
HochschudozentIn mit einschlägiger Expertise	8,8	9,4	5,2	5,2	11,0	12,7	6,8	4,8
LehrerIn, die/der modellhaft die Praxis präsentieren kann	45,4	38	46,6	43,1	49,3	38,1	31,8	31,0
VertreterIn von Einrichtungen (z. B. Medienzentrum)	5,9	7,7	3,4	3,4	5,9	9,0	9,1	9,5
Egal	37,0	41,9	39,7	43,1	31,6	38,8	50,0	50,0
*Zeitlicher Rahmen (χ*^*2*^*(68)* *=* *75,89, p* *=* *0,239 | χ*^*2*^*(50)* *=* *61,55, p* *=* *0,127)*								
Vormittag	52,8	51,7	57,1	55,3	54,9	52,5	42,3	44,4
Nachmittag	40,2	33,1	36,4	20,0	43,8	39,7	36,0	29,2
Ganztägig	62,5	54,1	52,9	44,1	64,3	60,2	69,2	46,2
Mehrtägige (Übernachtung zu Hause)	19,1	16,7	7,7	14,8	20,3	14,3	25,0	25,0
Mehrtägige (mit Übernachtung)	8,8	6,3	11,1	7,4	9,5	4,8	4,5	8,7
Fortbildungsreihe	12,3	13,7	14,8	14,3	10,9	12,1	13,6	17,4
Egal	29,2	27,3	30,0	32,1	25,7	22,7	37,9	33,3
*Zeitpunkt (χ*^*2*^*(26)* *=* *27,00, p* *=* *0,410 | χ*^*2*^*(22)* *=* *13,49, p* *=* *0,919)*								
Unterrichtszeit	72,7	69,3	82,8	72,4	73,5	73,5	68,2	65,9
Ferien	8,8	5,5	6,9	3,4	7,4	5,1	15,9	9,1
Wochenende	7,1	5,0	6,9	3,4	6,6	5,1	9,1	6,8
Egal	21,4	22,3	19,0	20,7	21,3	22,1	25,0	25,0
*Veranstaltungsort (****χ***^***2***^***(12)*** ***=*** ***24,01*, *p*** ***=*** ***0,020*** *| χ*^*2*^*(12)* *=* *20,30, p* *=* *0,062)*								
In der eigenen Schule	42,0	39,7	41,4	41,4	46,3	41,8	29,5	31,0
In (einer) anderen Schule(n) im lokalen Bereich	9,2	10,3	8,6	5,2	11,8	13,4	2,3	7,1
Hotel mit Tagungsraum	3,8	3,0	5,2	5,2	1,5	0,7	9,1	7,1
Hochschule	1,3	1,3	1,7	1,7	1,5	1,5	0,0	0,0
Medienzentrum	6,7	3,4	5,2	3,4	8,1	3,0	4,5	4,8
Egal	35,3	40,2	32,8	36,2	30,1	38,8	54,5	50,0
*Teilnehmendenkreis (χ*^*2*^*(10)* *=* *12,10, p* *=* *0,218 |* ***χ***^***2***^***(10)*** ***=*** ***28,34*, *p*** ***=*** ***0,002****)*								
Kollegiumsintern	15,5	19,7	10,3	12,1	17,6	23,3	15,9	19,0
In Teilkollegien auf freiwilliger Basis	14,7	11,2	22,4	24,1	15,4	9,0	2,3	0,0
Mit LehrerInnen aus versch. Schulen derselben Schulform	28,6	21,5	25,9	17,2	27,2	21,1	36,4	28,6
Schulformübergreifend	5,9	6,0	5,2	6,9	5,9	6,0	6,8	4,8
Egal	34,0	40,3	32,8	34,5	33,1	40,6	38,6	47,6
*Arbeitsform (χ*^*2*^*(286)* *=* *281,61, p* *=* *0,562 | χ*^*2*^*(232)* *=* *246,95, p* *=* *0,239)*								
Referat mit Diskussion	23,5	23,5	27,6	25,9	18,4	20,6	34,1	29,5
Informationsvermittlung mittels Medien (z. B. Film, Video, Buchtexte)	48,7	47,5	44,8	43,1	49,3	48,5	52,3	50,0
Kleingruppenarbeit	37,4	36,6	34,5	34,5	39,7	38,2	34,1	34,1
Erarbeitung von Unterrichts-materialien/-einheiten	52,1	48,3	51,7	46,6	54,4	50,7	45,5	43,2
Sichtung und Bewertung vorh. Unterrichtsmaterialien	46,2	42,4	48,3	46,6	45,6	41,2	45,5	40,9
Unterrichtshospitation	20,6	16,8	17,2	20,7	19,1	14,7	29,5	18,2
Exkursionen (z. B. Modellschulen)	20,6	12,6	22,4	15,5	16,9	11,0	29,5	13,6
Durchführung von Kommunikations‑, Plan‑, Rollenspielen u. ä.	9,2	12,6	12,1	17,2	9,6	11,0	4,5	11,4
Erprobung von Unterrichtskonzepten durch Eigentätigkeit	41,6	33,6	37,9	37,9	44,9	31,6	36,4	34,1
Blended learning	23,9	11,8	15,5	12,1	25,0	11,0	31,8	13,6
Ausschließlich online stattfindende Kurse	7,1	4,6	3,4	1,7	8,1	5,1	9,1	6,8
Egal	8,8	13,9	6,9	8,6	7,4	14,7	15,9	18,2

*Signifikante Ergebnisse sind fett gedruckt*

*FW* = Fortbildungswunsch, *DA* = digital Abseitsstehende, *DM* = digital Mithaltende, *DV* = digital Vorreitende

Hinsichtlich des *Niveaus* der Fortbildungen (Tab. 4) wünschten Abseitsstehende sich vor allem Grundlagenangebote. In der Gruppe der Mithaltenden wurden dagegen neben den grundlegenden Angeboten auch in fast ähnlichem Umfang vertiefende Fortbildungsangebote gewünscht. In der Gruppe der Vorreitenden sind dagegen Expertenangebote mehr als in den anderen beiden Gruppen gefragt, während sie sich vertiefende Angebote in ähnlichem Umfang wie die Mithaltenden wünschen und nur etwa ein Drittel nach grundlegenden Angeboten sucht. Die Unterschiede hinsichtlich des gewünschten Angebotsniveaus zwischen den Gruppen sind dabei signifikant.

Betrachtet man die präferierte *Expertise der Referenten*, ergab sich zwischen den Profilen kein erkennbarer Unterschied. Dies gilt auch im Hinblick auf den *zeitlichen Rahmen* und *Zeitpunkt* der Angebote sowie deren *Veranstaltungsort*. Im Hinblick auf den gewünschten *Teilnehmendenkreis* ähnelten sich die drei Gruppen ebenfalls in ihren Angaben. Ein leichter Unterschied bildete sich insofern ab, dass Abseitsstehende eher weniger Fortbildungen kollegiumsintern abhalten wollen, dafür aber häufiger die Option einer Teilnahme in Teilkollegien auf freiwilliger Basis präferierten als die anderen beiden Gruppen. Dieser Unterschied wurde jedoch nur beim zweiten Wunsch deutlich. Im Hinblick auf die präferierten *Arbeitsformen* ähnelten sich wiederum die Wünsche der Mitglieder aller drei Profile.

## Diskussion

### Zusammenfassung der Ergebnisse

Der vorliegende Beitrag untersucht, welche Präferenzen Lehrkräfte hinsichtlich der Inhalte und Gestaltungsmerkmale von Fortbildungen zu digitalen Medien äußern und welche Ableitungen sich in der Gesamtschau mit empirischen Befunden zum Thema ergeben. Eine LPA ermittelt dabei, ob der Einbezug von Personenmerkmalen, die im Zusammenhang mit digitalem Medieneinsatz stehen, eine sinnvolle Differenzierung für möglichst passgenaue Ableitungen zulässt.

(1a) Die gesammelten Angaben zeigen, dass die inhaltlichen Wünsche im Bereich digitaler Medien vielfältig sind. Auch wurde beispielsweise sichtbar, dass nach den am häufigsten genannten Grundlagenschulungen zu digitaler Mediennutzung (z. B. didaktische Konzepte) vor allem der Wunsch nach spezifischen Angeboten zu Hardware (z. B. interaktive Whiteboards) und Software (z. B. Lernapps, Lernplattformen) geäußert wurde, die bereits an vielen Schulen zumindest technisch realisiert sein dürfte. Dieser Befund bestärkt noch einmal die Forderungen nach einer ganzheitlichen Begleitung bei der Implementation digitaler Medien, die auch die Didaktik von digitalisierten Maßnahmen an Schulen mit einschließt (BMBF 2019). Dies spiegelt aber auch frühere Ergebnisse wider, nach denen vor allem grundlegende Inhalte im Zusammenhang mit digitalen Medien gewünscht werden (z. B. Gysbers 2008). Im Vergleich zu der überproportional häufig genannten Themenwunschkategorie „Einsatz digitaler Medien (Computer, Tablet, Smartphone etc.) im Unterricht oder für Hausaufgaben“ waren die Themenwünsche der Lehrkräfte im Länderindikator 2016 (Kammerl et al. 2016) eher gleichverteilt über die dort genutzten Kategorienvorschläge. Dieser Unterschied kann auch mit den in dieser Studie durchgeführten Ausdifferenzierung des Kategoriensystems einhergehen. Analog zu den Studienergebnissen von Crompton und Burke (2020) fanden weiterführende Funktionen und Potenziale digitaler Medien, wie beispielsweise das elektronische Prüfen oder adaptive Differenzierungsmöglichkeiten, dagegen selten Erwähnung. Lehrkräfte sollten daher auch in digitalen Grundlagenfortbildungen dazu angeregt werden, verfügbare Technik nicht nur als Ersatz für analoge Medien zu nutzen. Dadurch kann das Potenzial digitaler Medien bei didaktisch sinnvoller Integration durch die Gestaltung neuartiger Lehr-Lernsituationen verdeutlicht werden.

(1b) Die Ergebnisse zu Rahmenbedingungen und Gestaltungsoptionen der Fortbildungen ähnelten denen früherer Studien: Lehrkräfte präferierten unabhängig vom Inhalt Dozierende, die selbst Lehrkräfte sind und aus der Praxis berichten können sowie halb- oder eintägige Veranstaltungen, die innerhalb der Woche und außerhalb der Schulferien stattfinden (Krille 2020). Die eigene Schule (und somit eine kurze Anreise) war besonders beliebt. Allerdings gab es durchgehend auch einen recht großen Anteil an Angaben, dass die konkrete Gestaltung nicht relevant sei, so dass offenbar die inhaltliche Gestaltung im Mittelpunkt steht. Problematisch ist insgesamt, dass die gewünschten Fortbildungsmerkmale in dieser Form eher konträr zu den Befunden hinsichtlich wirksamer Lehrkräftefortbildung stehen. Die von den Lehrkräften „zusammengestellten“ Fortbildungen sind eher kurz und geben kaum Raum für Reflexion. Wie auch in früheren Studien (z. B. Florian 2008) wurden ausschließlich online stattfindende Kurse, aber auch Blended-Learning-Formate kaum präferiert.

(2a & b) Die zweite Forschungsfrage adressierte, inwiefern sich die im Zusammenhang mit digitalen Medien stehenden Wünsche zu Fortbildungsthemen und -gestaltung in Abhängigkeit von Personenmerkmalen unterscheiden. Die Profilanalyse konnte analog zu bisherigen Studien (z. B. Klassen zu Lehrerprofessionalisierungstypen, Drossel und Eickelmann 2018) zeigen, dass sich personenbezogene Variablen wie der Medieneinsatz und digitale Wissensfacetten so in Personengruppen bündeln ließen, dass sie eine implizite Erwartung in der Aufgeschlossenheit und den Umgang mit digitalen Medien abbilden. Wir konnten eine sich selbst als kompetent einschätzende Gruppe (die Vorreitenden), eine sich als durchgehend weniger kompetent einschätzende Gruppe (die Abseitsstehenden) sowie eine eher indifferente Gruppe zwischen den beiden Extremen (Mithaltende) identifizieren. Besonders deutlich unterschieden sich die beiden Extremgruppen in ihrer Einschätzung ihres technologischen, ebenso wie im technologisch-pädagogischen Wissens und in der Selbstwirksamkeit im Umgang mit digitalen Medien. Dieses Ergebnis ist insbesondere vor dem Hintergrund der aktuellen Anforderungen an Lehrkräfte für die Gruppe der Vorreitenden ebenso erfreulich, wie es für die Gruppe der Abseitsstehenden besorgniserregend ist: Es bleibt zu vermuten, wie wenig erfolgreich und zufriedenstellend für Lehrkräfte und Schülerinnen und Schüler ein durch digitale Medien geprägter Schulalltag durch letztere sein dürfte.

(2c) Bei vertiefenden Analysen zu Unterschieden zwischen den Gruppen zeigte sich ein erwartungskonformer Unterschied in den bisher besuchten Fortbildungsveranstaltungen zu digitalen Medien. Digital Abseitsstehende besuchten in den letzten zwei Jahren signifikant weniger solcher Veranstaltungen, wobei der Unterschied nicht auf eine generell niedrigere Fortbildungsteilnahme zurückzuführen ist. Auch die Unterschiede in den Hinderungsgründen spiegeln das mangelnde Interesse an der Arbeit mit digitalen Medien im Unterricht wider (z. B. höhere Priorität anderer Themen, kein Interesse an Thematik oder zukünftigem Einsatz). Vor dem Hintergrund, dass in Deutschland die Wahl von Fortbildungsthemen in erster Linie bei der Lehrkraft liegt und diese Wahl stark von intrinsischen Faktoren wie dem Interesse abhängt (Krille 2020), wird deutlich, wie schwer es ist, diese Zielgruppe mit Fortbildungsveranstaltungen zu digitalen Medien zu erreichen. Im ungünstigsten Fall ist hier zu befürchten, dass sich über eine ausbleibende Beschäftigung mit der Thematik die Schere zwischen den interessierten und kompetenten Lehrkräften und den Lehrkräften mit einer vermeidenden Haltung noch stärker aufmacht: Den kompetenten Lehrkräften dürfte es leichter fallen, sich auch mit Neuerungen zu beschäftigen und gezielt Unterstützungsangebote wahrzunehmen, während andere durch fehlende selbstwirksamkeitsrelevante Erlebnisse auf der einen und die steigende Zahl digitaler Optionen auf der anderen Seite, potenziell noch weiter von Fortbildungsangeboten Abstand nehmen. Am ehesten sollte eine Ansprache gelingen, indem man dem Wunsch der Gruppe nachkommt, Einführungsveranstaltungen auf einem Grundlagenniveau zu konzipieren. Die Veranstaltungen sollten einen geschützten Rahmen bieten, in dem diese Teilnehmenden Fragen ohne die Besorgnis stellen können, durch ihr geringes Vorwissen unangenehm vor Kolleginnen und Kollegen dazustehen. Im Hinblick auf die sonstigen Rahmenbedingungen ergab die Befragung keine Unterschiede zwischen den gefundenen Profilen.

### Limitationen

Das Medium der Onlinebefragung könnte eine Selbstselektivität dahingehend begünstigt haben, dass digital weniger Interessierte erst gar nicht teilgenommen haben. Hier ist zwar anzuführen, dass an einzelnen Schulen ganze Kollegien teilgenommen haben und auch die Ergebnisse auf eine Heterogenität hinweisen, jedoch wären bei Folgeuntersuchungen Vollerhebungen aller beteiligten Kollegien wünschenswert. Zudem wurden nur Schulen mit gymnasialer Oberstufe aus dem Raum Frankfurt befragt. Für eine stärkere Generalisierbarkeit der Ergebnisse wären bundesweite Erhebungen unter Berücksichtigung aller Schularten günstig. Eine weitere Limitation ergibt sich aus dem Datenmaterial der offenen Angaben für die Kategorisierung der Fortbildungswünsche. Da die Angaben häufig nur aus kurzen Stichworten bestanden, war es teilweise schwer zu erkennen, was die Fortbildung beinhalten soll. Zum Beispiel wurde nicht immer klar ersichtlich, ob eine Kompetenz oder Wissen selbst erworben werden soll oder es darum geht, wie man diese an Schülerinnen und Schüler vermittelt (z. B. Film- oder Bildbearbeitung).

Auch ergibt sich durch das offene Antwortformat zwar prinzipiell die Möglichkeit, über eine Liste von Antwortoptionen hinaus eigene Wünsche zu formulieren, gleichzeitig sollte berücksichtigt werden, dass Personen mit wenigen Erfahrungen auch eine weniger differenzierte Informationsbasis besitzen, aus denen sich ihre Antworten speisen.

Ausführlichere Befragungsformate und insbesondere weitere qualitative Verfahren wie Interviewstudien wären hier eine wünschenswerte Ergänzung. Die Befragung fand zudem vor den Onlineunterrichtsphasen und Schulschließungen aufgrund der COVID-19-Pandemie statt. Es ist davon auszugehen, dass diese einen wesentlichen Einfluss auf die aktuellen Wünsche und die Präferenzen zur Gestaltung von Fortbildungen zu digitalen Medien hat. Dennoch geben die vorliegenden Ergebnisse einen Einblick in digitalisierungsbezogene Wissensfacetten und Mediennutzung der Lehrkräfte und lassen Rückschlüsse und Empfehlungen auf die Gestaltung von Fortbildungen zu digitalen Medien zu. Zur Generalisierbarkeit der in der LPA gefundenen Profile konnte diese zwar durch die Prüfung von Annahmen innerhalb der vorliegenden Stichrobe bestätigt werden, dennoch wäre es wünschenswert eine ergänzende Validierung an einer weiteren Stichprobe (Hartig et al. 2008) durchzuführen.

## Implikationen

Kompetenzen im Umgang mit digitalen Medien in Lehr-Lernszenarien werden durch zunehmende gesellschaftliche Digitalisierungsprozesse immer relevanter (KMK 2016). Diese Relevanz wird vor allem für Lehrkräfte gerade in Phasen von vermehrtem Distanzlernen und Onlineunterricht deutlich (Eickelmann und Gerick 2020). Ein breites Angebot an Fortbildungsformaten sollte als Unterstützungsstruktur für Lehrkräfte den notwendigen Kompetenzerwerb ermöglichen. Die nahezu flächendeckende Notwendigkeit, sich durch den mit der COVID-19-Pandemie einhergehenden Digitalisierungsschub mit digital gestütztem Lehren und Lernen zu beschäftigen, stellt eine prinzipiell günstige Ausgangsbasis dar: Es ist davon auszugehen, dass nicht nur einschlägig vorgebildete und interessierte Personen Angebote zu digitalen Themen in Betracht ziehen (vgl. Neigungshypothese, Richter 2011). Eine ernstgenommene Evidenzbasierung legt dabei nicht nur den Bezug auf einschlägige Kompetenzmodelle, wissenschaftlich aktuelle und relevante Inhalte, wie auch die Berücksichtigung von Erkenntnissen aus der Wirksamkeitsforschung zu Fortbildungen nahe. Es sollten zudem auch Bedarfe und Wünsche der Zielgruppe lokalisiert werden, um auf diese passgenau eingehen zu können und die potenzielle Bereitschaft zur Teilnahme zu erhöhen. Dadurch sollten Fortbildungsangebote für die anvisierte Zielgruppe im besten Fall als sinnvolles Unterstützungsangebot wahrgenommen werden, um die eigene Professionalisierung für einen reflektierten, sicheren und zielgerichteten Umgang mit digitalen Medien bei der Unterrichtsvor- und -nachbereitung zu fördern. Im Folgenden sollen einige Empfehlungen für die Konzeption von Fortbildungsmaßnahmen zusammengefasst werden:

1. Lehrkräfte mit didaktischem Rüstzeug für die Techniknutzung ausstatten

Die Ergebnisse der vorliegenden Studie weisen einmal mehr darauf hin, dass auch bei bereits länger in Schulen etablierten digitalen (z. B. Smartboards) oder in der Breite angeschafften Endgeräten (z. B. Tablets) ein Fortbildungsbedarf besteht. Um den lernförderlichen Einsatz digitaler Endgeräte in Schule und Unterricht bestmöglich zu unterstützen, ist die Bereitstellung technischer Infrastruktur nicht hinreichend. Vielmehr müssen Lehrkräfte wie bereits in aktuellen Förderprogrammen (BMBF 2019) und Strategien zur Digitalisierung (KMK 2016) gefordert, neben funktionierender Technik auch mit didaktischem Rüstzeug ausgestattet werden (siehe auch Walitzek 2021).

2. Digitalisierungsspezifische Themen in praktischen Anwendungsszenarien bündeln

Da die zur Verfügung stehende Zeit für die Teilnahme an Fortbildungen bei Lehrkräften begrenzt ist und viele relevante Themen konkurrieren, kann die Vielzahl der genannten Fortbildungswünsche zum Einsatz digitaler Medien kaum realistisch adressiert werden. Daher sollten mögliche digitalisierungsspezifische Themen wie beispielsweise das Verhalten bei technischen Problemen, Datenschutz und -sicherheit oder aber auch der Umgang mit Ablenkungen der Schülerinnen und Schüler durch den Einsatz digitaler Medien lokalisiert und gezielt in Fortbildungen genutzt werden, beispielsweise als exemplarische Anwendungsszenarien. Dies kommt der von Lehrkräften genannten Präferenz eines Praxisbezuges (Krille 2020) entgegen und hat zudem das Potenzial durch den praktischen Schulbezug von Fortbildungen deren Transferwahrscheinlichkeit zu erhöhen (z. B. Vigerske 2017).

3. Potenziale digitaler Medien für Fortbildungen nutzen

Um der thematischen Vielfalt an Fortbildungsinhalten und den unterschiedlichen Expertisegraden von Lehrkräften zu begegnen, sollte künftig im Bereich der Fortbildungen zu digitalen Medien das orts- und zeitunabhängige sowie adaptive Potenzial von digitalen Formaten verstärkt genutzt werden (Eickelmann und Drossel 2020b). Analysen des Fortbildungsangebots zeigen, dass diese Potenzial bisher von Fortbildungsanbietern kaum genutzt wird (Engec und Endberg 2020). Und auch wenn diese vor der COVID-19 Pandemie von Lehrkräften kaum präferiert wurden, bieten sich (nicht nur) in Zeiten von Kontaktbeschränkungen Selbstlernkurse oder Web-Based Trainings an, die im besten Fall als Open Educational Ressources (OER) breit nutzbar gemacht werden können. Aber auch synchrone webbasierte oder Blended-Formate, die virtuelle Elemente mit Präsenzterminen kombinieren, schaffen Angebote, die regional unabhängig genutzt werden könnten und trotzdem dem Wunsch nach kollegialem Austausch (Krille 2020) entgegenkommen. Durch ein solch offenes Fortbildungsangebot könnten Lehrkräfte ihre Kompetenzentwicklung selbstreguliert und bedarfsorientiert gestalten. Durch die auch in der vorliegenden Studie gezeigte Zurückhaltung vieler Lehrkräfte gegenüber ausschließlich virtuell stattfindenden Angeboten und Blended-Learning-Modellen sollte die Ansprache von Schulen und Lehrkräften besonders sorgfältig und mit einem Fokus auf einfache Zugänglichkeit und zu erwartende Mehrwerte erfolgen.

4. Motivationale Faktoren adressieren

Um die Lehrkräfte entsprechend ihres Expertisegrades nicht zu über- oder unterfordern und damit das Fernbleiben von Fortbildungen nicht zu befördern, sollten relevante Themenfelder von Fortbildungen zu digitalen Medien mit verschiedenen Anforderungsniveaus angeboten werden. Eine Antizipation der identifizierten Profile kann hier eine hilfreiche Heuristik darstellen: Ein angemessenes Anspruchsniveau ermöglicht es Individuen, (Teil‑)Ziele bei neuen Lerngegenständen zu erreichen und so die Selbstwirksamkeitserwartung zu steigern (Schwarzer und Jerusalem 2002). Eine Steigerung der Selbstwirksamkeit sollte wiederum mit einer höheren (künftigen) Teilnahmewahrscheinlichkeit einhergehen (z. B. Richter et al. 2013). Wie auch bei generellen Fortbildungsthemen wurden in der vorliegenden Befragung ein fehlender Bezug zur Unterrichtspraxis und Transfermöglichkeiten als Hinderungsgründe aufgeführt – insbesondere von Personen mit geringerem Vorwissen und geringerer Selbstwirksamkeitserwartung. Fortbildungen sollten daher entsprechend Bezüge zu Schule und Unterricht herstellen, beispielsweise auch durch die Umsetzung fachdidaktischer Angebote, und dies in der Fortbildungsankündigung bereits deutlich machen, um die Motivation zur Fortbildungsteilnahme zu erhöhen.

5. Kollegialen Austausch zu Themen der Digitalisierung fördern

Der kollegiale Austausch von Lehrkräften hat sich bereits in vergangenen Studien (z. B. Drossel et al. 2019; Gräsel et al. 2006) als Prädiktor für die tägliche Integration digitaler Medien im Unterricht erwiesen. Diese Ergebnisse und der Wunsch von Lehrkräften nach kollegialem Austausch, wie er in der vorliegenden Studie ebenfalls gezeigt werden konnte, sollte durch die Gestaltung von Fortbildungen mit entsprechenden Formaten befördert werden. Hierfür eignet sich die Integration von kooperativen Elementen in die Fortbildungsgestaltung, die zu einem systematischen Austausch anregen. Dies könnte beispielsweise durch die gemeinsame Erarbeitung von digitalen Unterrichtskonzepten, Peer-Feedback auf digitale Unterrichtsmaterialien oder einer aus der Fortbildung ausgelagerten kollegialen Hospitation von (digitalen) Unterrichtseinheiten umgesetzt werden. Solche Formate hätten somit nicht nur ein motivationales Potenzial, sondern können erfolgreiche Konzepte von digitalem Medieneinsatz auch über die eigenen Schulgrenzen hinaus befördern.

6. Fortbildungszentren errichten

Auch wenn bundesweite Fortbildungsverpflichtungen bisher nicht in allen Ländern positive Zusammenhänge mit der tatsächlichen Teilnahme an Angeboten zeigen (Kuschel et al. 2020), könnten obligatorische Basisfortbildungen eine Möglichkeit zur Sicherung von Lehrqualität bei der Nutzung digitaler Medien darstellen. Da Angebote dieser Art kontinuierlich und breit zugänglich vorgehalten werden müssten, bietet sich die Realisierung über Fortbildungszentren an, die auch der absehbaren Realität von Fortbildung als Daueraufgabe in der Lehrkräftebildung entsprächen (vgl. auch BMBF 2020). Solche Fortbildungszentren sind nicht nur als Schnittstelle zu den Schulen und Lehrkräften, sondern auch zu wissenschaftlichen Institutionen denkbar.

Letztlich sollte es das Ziel sein, nicht nur Lehrkräfte für das Thema der digitalen Lehrerkräftefortbildungen zu gewinnen, die der zunehmenden Digitalisierung von Schule und Unterricht offen gegenüberstehen. Eine kritische wissenschaftliche Begleitung in Form von Lehrkräftebefragungen und Evaluation der Wirksamkeit von Fortbildungen stellt daher künftig weiterhin eine sinnvolle Maßnahme und herausfordernde Aufgabe dar, um Fortbildungen in diesem Bereich attraktiv und wirksam für eine größtmögliche Gruppe von Lehrkräften zu gestalten.
